# The Arab region’s contribution to global COVID-19 research: Bibliometric and visualization analysis

**DOI:** 10.1186/s12992-021-00690-8

**Published:** 2021-03-25

**Authors:** Sa’ed H. Zyoud

**Affiliations:** 1 Department of Clinical and Community Pharmacy, College of Medicine and Health Sciences, An-Najah National University, Nablus, 44839 Palestine; 2 Clinical Research Centre, An-Najah National University Hospital, Nablus, 44839 Palestine

**Keywords:** Arab world, Bibliometric, COVID-19, Novel coronavirus, Scopus

## Abstract

**Background:**

At the global level and in the Arab world, particularly in low-income countries, COVID-19 remains a major public health issue. As demonstrated by an incredible number of COVID-19-related publications, the research science community responded rapidly. Therefore, this study was intended to assess the growing contribution of the Arab world to global research on COVID-19.

**Methods:**

For the period between December 2019 and March 2021, the search for publications was conducted via the Scopus database using terms linked to COVID-19. VOSviewer 1.6.16 software was applied to generate a network map to assess hot topics in this area and determine the collaboration patterns between different countries. Furthermore, the research output of Arab countries was adjusted in relation to population size and gross domestic product (GDP).

**Results:**

A total of 143,975 publications reflecting the global overall COVID-19 research output were retrieved. By restricting analysis to the publications published by the Arab countries, the research production was 6131 documents, representing 4.26% of the global research output regarding COVID-19. Of all these publications, 3990 (65.08%) were original journal articles, 980 (15.98%) were review articles, 514 (8.38%) were letters and 647 (10.55%) were others, such as editorials or notes. The highest number of COVID-19 publications was published by Saudi Arabia (*n* = 2186, 35.65%), followed by Egypt (*n* = 1281, 20.78%) and the United Arab Emirates (UAE), (*n* = 719, 11.73%). After standardization by population size and GDP, Saudi Arabia, UAE and Lebanon had the highest publication productivity. The collaborations were mostly with researchers from the United States (*n* = 968), followed by the United Kingdom (*n* = 661). The main research lines identified in COVID-19 from the Arab world are related to: public health and epidemiology; immunological and pharmaceutical research; signs, symptoms and clinical diagnosis; and virus detection.

**Conclusions:**

A novel analysis of the latest Arab COVID-19-related studies is discussed in the current study and how these findings are connected to global production. Continuing and improving future collaboration between developing and developed countries will also help to facilitate the sharing of responsibilities for COVID-19 in research results and the implementation of policies for COVID-19.

## Background

Coronavirus disease 2019 (COVID-19) first came to light in December 2019 with the appearance of viral pneumonia cases in Wuhan City, Hubei Province, China [1, 2]. COVID-19 has produced heavy burdens and brought enormous global public health challenges. In the first 6 months of the pandemic, the novel coronavirus caused more than 1 million deaths and enormous economic and social upheavals worldwide [3, 4]. The World Health Organization (WHO) declared the COVID-19 outbreak a Public Health Emergency of International Significance on 30 January 2020 and described the COVID-19 epidemic as a pandemic 6 weeks later.

As of 9 March 2021, more than 116.5 million cases of COVID-19 and more than 2.5 million deaths from the disease have been reported worldwide [5]. A study conducted by Al-Kindi indicated that the rate of COVID-19 infection is higher in the most populated areas [6]. COVID-19 has a high death rate in hospitalized patients due to respiratory failure, with certain patients needing mechanical ventilation [7]. Some drugs have been available, including antimalarials (e.g. chloroquine), antivirals (e.g. lopinavir/ritonavir, remdesivir), anti-inflammatories (e.g. dexamethasone) and monoclonal antibodies (e.g. tocilizumab) but their actual effect on the course of the infection was obscure at the start of the pandemic [8].

The emerging global threat of COVID-19 has contributed to an explosion of publications on coronaviruses. COVID-19-related publications have been published increasingly and the findings of scientific studies are continuously emerging with the collaborative efforts of researchers and clinicians around the world [9–14]. As of 8 March 2021, 110,839 published articles on COVID-19 were included in PubMed [15].

As the number of scientific publications rapidly increases, it is important to dissect the variables that lead to highly impactful publications. Bibliometrics, along with visualization techniques, have been reported to be helpful in evaluating research output for emerging infectious disease outbreaks [16–24].

It can be seen that the existing literature has revealed some important issues in the field of COVID-19, such as important documents, co-citation networks [10–12, 25–32] and the development status of COVID-19 in a specific field, such as COVID-19 in the environment [33], registered clinical trials on the COVID-19 pandemic [13], traditional Chinese medicine for COVID-19 [34] and business and management during the COVID-19 pandemic [35]. Previous publications on COVID-19 primarily evaluated international studies’ research performance and paid less attention to the research framework of COVID-19 in the Arab world. In other words, in the Arab world there is a lack of bibliometric studies on COVID-19 investigating the research performance in a quantitative method, and the connection between hot research topics has not been clearly disclosed. Therefore, the aim of this analysis was to assess the volume and impact of the Arab scientific output among the COVID-19 publications indexed in Scopus. Consequently, this study’s main subject is the collaboration network, along with existing research topics and hotspots that need to be further studied. The findings could help to identify more effective approaches to future research in the funding, planning, implementation and networking of quality and sustainability-based research.

## Methods

All publications indexed in Scopus as COVID-19 were downloaded and analysed by bibliometric methods. The Scopus database offers a wider range of journals compared to PubMed and Web of Science [36]. Furthermore, it has more non-English scientific journals than Web of Science, which is important because no language restriction was applied in the current research. The Scopus database provides comprehensive, multidisciplinary citation data and is considered one of the primary data sources for bibliometric analysis [36–38]. In addition, Scopus data can be easily exported to Microsoft Excel or third-party software such as VOSviewer for further analysis and mapping.

The search took place in March 2021 and all publications published before 8 March 2019 were evaluated. The finalized search string with COVID-19 primary emphasis and keywords used in the TITLE-ABSTRACT-KEYWORDS fields [11, 25, 32, 39, 40] is as follows: “coronavirus 2019” or “COVID 19” or “coronavirus disease 2019” or “2019 novel coronavirus” or “2019-novel CoV” or “COVID 2019” or “2019 ncov” or “COVID19” or “nCoV-2019” or “nCoV2019” or “nCoV 2019” or “COVID-19” or “Severe acute respiratory syndrome coronavirus 2” or “2019-ncov” or “SARS-CoV-2”. All 22 Arab countries [41] were used as country keys in this study, accompanied by terms related to COVID-19 (Table 1).

**Table 1:** Ranking and contribution of Arab countries in research on COVID-19

Rank	Country	Number of publication	%	Population by milliun	GDP by billion	AI	AI Rank
1st	Saudi Arabia	2186	35.65	35	800	49.97	1st
2nd	Egypt	1281	20.89	100	350	4.48	6th
3rd	UAE	719	11.73	10	450	32.36	2nd
4th	Morocco	431	7.03	35	120	1.48	9th
5th	Jordan	430	7.01	10	45	1.94	8th
6th	Qatar	402	6.56	3	220	9.19	5th
7th	Iraq	357	5.82	40	250	1.25	11th
8th	Lebanon	347	5.66	7	55	15.62	3rd
9th	Tunisia	222	3.62	12	50	0.76	16
10th	Oman	205	3.34	5	80	0.92	12th
11th	Kuwait	203	3.31	4	180	14.89	4th
12th	Algeria	135	2.20	45	220	0.84	15th
13th	Bahrain	110	1.79	1.6	35	0.86	14th
14th	Sudan	101	1.65	40	80	0.42	17th
15th	Palestine	86	1.40	4.5	16	1.38	10th
15th	Yemen	86	1.40	30	40	3.87	7th
17th	LAJ	57	0.93	7	80	0.28	18th
18th	SAR	42	0.69	22	40	0.92	13th
19th	Somalia	14	0.23	14	1.1	0.03	20th
20th	Mauritania	10	0.16	4.5	8	0.04	19th
21st	Comoros	2	0.03	0.8	1.2	0.00	22nd
21st	Djibouti	2	0.03	0.9	3.5	0.02	21st

Abriviations: *AI* adjustment index, *GDP* gross domestic product, *LAJ* Libyan Arab Jamahiriya, *SAR* Syrian Arab Republic, *UAE* United Arab Emirates

The following formula was used to calculate an adjustment index (AI): AI = [Total number of publications for the country / GDP per capita of the country] × 1000, where GDP per capita is the country’s GDP divided by its population

The bibliometric parameters used to analyse the publications related to COVID-19 from Arab countries were: type of documents, publication output, journals, country and institutions, publication patterns, citation patterns and collaboration analysis. Furthermore, the research output of Arab countries was adjusted by using the adjustment index (AI) formula in relation to population size and gross domestic product (GDP) in 2019 [42]. The following formula was used to calculate the AI [43–45]: AI = [Total number of publications for the country / GDP per capita of the country] × 1000, where GDP per capita is the country’s GDP divided by its population.

The most commonly used terms and collaboration between countries were recognized by using the VOSviewer bibliometric software (version 1.6.16) [46], which made it possible to view the measured variables on scientific maps. The maps or clusters were generated by the union of terms or countries that have some connection between them, creating individual clusters and distinguished by similar colours. In term clusters, a frame labels each word. The frame size reflects the number of publications in the collection of selected papers for the term. Therefore, we decided to produce and visualize the network terms that were used in the title/abstract of publications to identify hot topics in this field.

## Results

The total number of COVID-19-related publications obtained by using COVID-19-related terms as a topic in the Scopus search engine (Title/Abstract/Keywords) without stating the name of any country was 143,975 publications, reflecting the overall global COVID-19 research output. Just 6131 (4.26% of the total global COVID-19 research output) publications were collected from the Arab countries using the methodology mentioned above: 3990 (65.08%) original journal articles, 980 (15.98%) review articles, 514 (8.38%) letters and 647 (10.55%) others, such as editorials or notes.

The country-by-country publication review found that the highest number of COVID-19 publications was published by Saudi Arabia (*n* = 2186, 35.65%), followed by Egypt (*n* = 1281, 20.78%) and the United Arab Emirates (UAE) (*n* = 719, 11.73%). In Table 1, the list of Arab countries is shown, ordered by AI based on the GDP per capita. Saudi Arabia is ranked first in both production and AI in these results. The UAE, Lebanon and Kuwait come in second, third and fourth, respectively. Lebanon and Yemen, on the other hand, rank third and seventh, respectively, according to the AI which are considered as lower GDP per capita than the other nations.

In addition, 3135 (51.13%) publications from collaborations with 138 non-Arab countries were recognized by the analysis. Table 2 shows the collaboration between Arab countries and the top 20 non-Arab countries in the research on COVID-19. These collaborations were mostly with researchers from the USA (*n* = 968, 15.79%), followed by the UK (*n* = 661, 10.78%), India (*n* = 550, 8.79%), Pakistan (*n* = 363, 5.92%) and Italy (*n* = 341, 5.56%).

**Table 2:** Collaboration between Arab countries and top 20 non-Arab countries in research on COVID-19

Ranking	Country	Number of publication	%
1st	United States	968	15.79
2nd	United Kingdom	661	10.78
3rd	India	550	8.97
4th	Pakistan	363	5.92
5th	Italy	341	5.56
6th	Canada	319	5.20
7th	China	306	4.99
8th	Australia	272	4.44
9th	France	250	4.08
10th	Germany	209	3.41
10th	Malaysia	209	3.41
12th	Spain	189	3.08
13th	Turkey	174	2.84
13th	Iran	160	2.61
15th	Switzerland	150	2.45
16th	South Africa	148	2.41
17th	Brazil	138	2.25
18th	Japan	129	2.10
18th	Netherlands	129	2.10
20th	South Korea	116	1.89

**Fig. 1:**
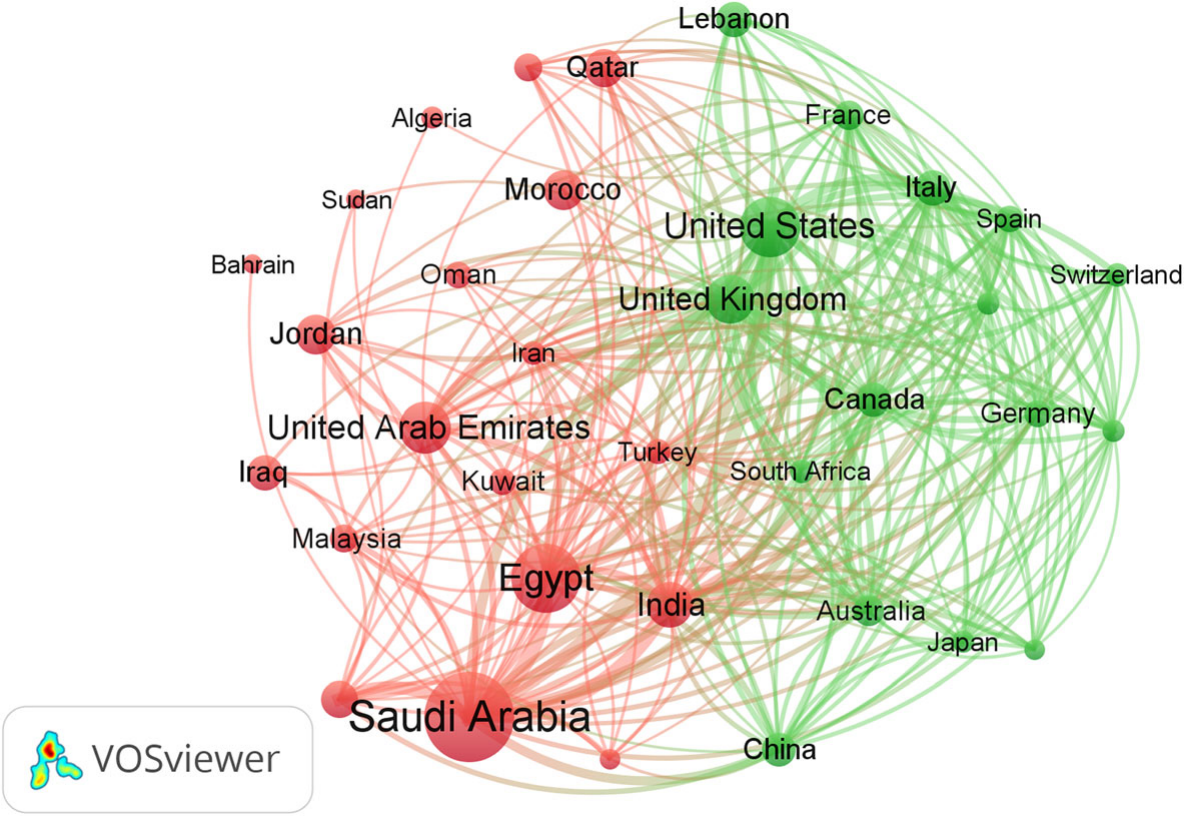
Network visualization map of Arab international research collaboration among countries with minimum research output of 100 documents on COVID-19-related publications from the Arab world. The map was created using VOSviewer software version 1.6.16

Figure 1 illustrates a visualization network of cooperation between Arab countries and also between Arab and non-Arab countries, created using the VOSviewer visualization software. The USA and the UK are at the centre of cooperation and have the most substantial collaboration with Arab countries.

A total of 25,562 institutions contributed to 6131 publications on COVID-19. Table 3 shows the top ten institutions with the largest number of publications on COVID-19 from Arab countries. The *King Saud University* (*n* = 429 publications) ranked first, followed by *King Abdulaziz University* (*n* = 308 publications) and *Cairo University* (*n* = 278 publications). In addition, Saudi Arabia accounted for four of the top ten institutions, indicating that the country has many outstanding research groups in this area.

**Table 3:** The top ten rankings of productive institutions from Arab countries

Ranking	Institution	Country	***n***	%
1st	*King Saud University*	Saudi Arabia	429	7.00
2nd	*King Abdulaziz University*	Saudi Arabia	308	5.02
3rd	*Cairo University*	Egypt	278	4.53
4th	*King Saud bin Abdulaziz University for Health Sciences*	Saudi Arabia	151	2.46
5th	*Imam Abdulrahman Bin Faisal university*	Saudi Arabia	142	2.32
6th	*Hamad Medical Corporation*	Qatar	140	2.28
7th	*Qatar University*	Qatar	136	2.22
8th	*University of Sharjah*	United Arab Emirates	132	2.15
9th	*Jordan University of Science and Technology*	Jordan	130	2.12
10th	*American University of Beirut*	Lebanon	123	2.01

The ten most influential peer-reviewed journals are presented in Table 4, representing approximately 8.45% of the total journals publishing scientific research in this field. The *International Journal of Environmental Research and Public Health* (1.16%), the *Pan African Medical Journal* (1.04%) and the *Journal of Biomolecular Structure and Dynamics* (0.91%) were ranked as the top three most influential journals, with 71, 64 and 56 publications, respectively. In the top ten productive journals the number of publications is not high, accounting for just 8.45% of all publications, which indicates that a wide variety of mainstream journals are available, providing more resources for the large research interest in this area.

**Table 4:** The top ten rankings of journals publishing COVID-19-related publications from the Arab world

Ranking	Journal	***n***	%	IF
1st	*International Journal of Environmental Research and Public Health*	71	1.16	2.849
2nd	*Pan African Medical Journal*	64	1.04	NA
3rd	*Journal of Biomolecular Structure and Dynamics*	56	0.91	3.301
4th	*Plos One*	55	0.90	2.740
5th	*International Journal of Infectious Diseases*	47	0.77	3.202
5th	*Results in Physics*	47	0.77	4.019
7th	*Frontiers in Public Health*	46	0.75	2.483
8th	*Medical Hypotheses*	45	0.73	1.375
9th	*Journal of Infection and Public Health*	44	0.72	2.447
10th	*Chaos Solitons snd Fractals*	43	0.70	3.764

IF is the impact factor for 2019 journals listed in Incites Journal Citation Reports, Clarivate Analytics

The research history of COVID-19 is short but dynamic. A total of 25,193 citations were obtained from publications on COVID-19. The *h*-index was 64 and, on average, each paper earned 4.11 citations. Table 5 shows the top 20 most cited articles in the field of COVID-19, with the citation counts ranging from 138 to 940 [47–66].

**Table 5:** The 20 most cited articles in the area of COVID-19 in Scopus from the Arab world

Ranking	Authors	Year	Source title	Cited by
1st	Hui et al. [47]	2020	*International Journal of Infectious Diseases*	940
2nd	Rodriguez-Morales et al. [48]	2020	*Travel Medicine and Infectious Disease*	642
3rd	Chu et al. [49]	2020	*The Lancet*	538
4th	Alhazzani et al. [50]	2020	*Intensive Care Medicine*	534
5th	Bedford et al. [51]	2020	*The Lancet*	380
6th	Phua et al. [52]	2020	*The Lancet Respiratory Medicine*	375
7th	COVIDSurg Collaborative [53]	2020	*The Lancet*	285
8th	Petrosillo et al. [54]	2020	*Clinical Microbiology and Infection*	259
9th	Elfiky [55]	2020	*Life Sciences*	227
10th	Xu et al. [56]	2020	*Viruses*	217
11th	Tahir ul Qamar et al. [57]	2020	*Journal of Pharmaceutical Analysis*	215
12th	Elfiky [58]	2020	*Life Sciences*	213
12th	Alhazzani et al. [59]	2020	*Critical Care Medicine*	213
14th	Al-Shamsi et al. [60]	2020	*Oncologist*	177
15th	Khailany et al. [61]	2020	*Gene Reports*	149
16th	Rabi et al. [62]	2020	*Pathogens*	147
17th	Meo et al. [63]	2020	*European Review for Medical and Pharmacological Sciences*	146
18th	Zumla et al. [64]	2020	*The Lancet*	141
19th	Bastard et al. [65]	2020	*Science*	138
19th	Ashour et al. [66]	2020	*Pathogens*	138

**Fig. 2:**
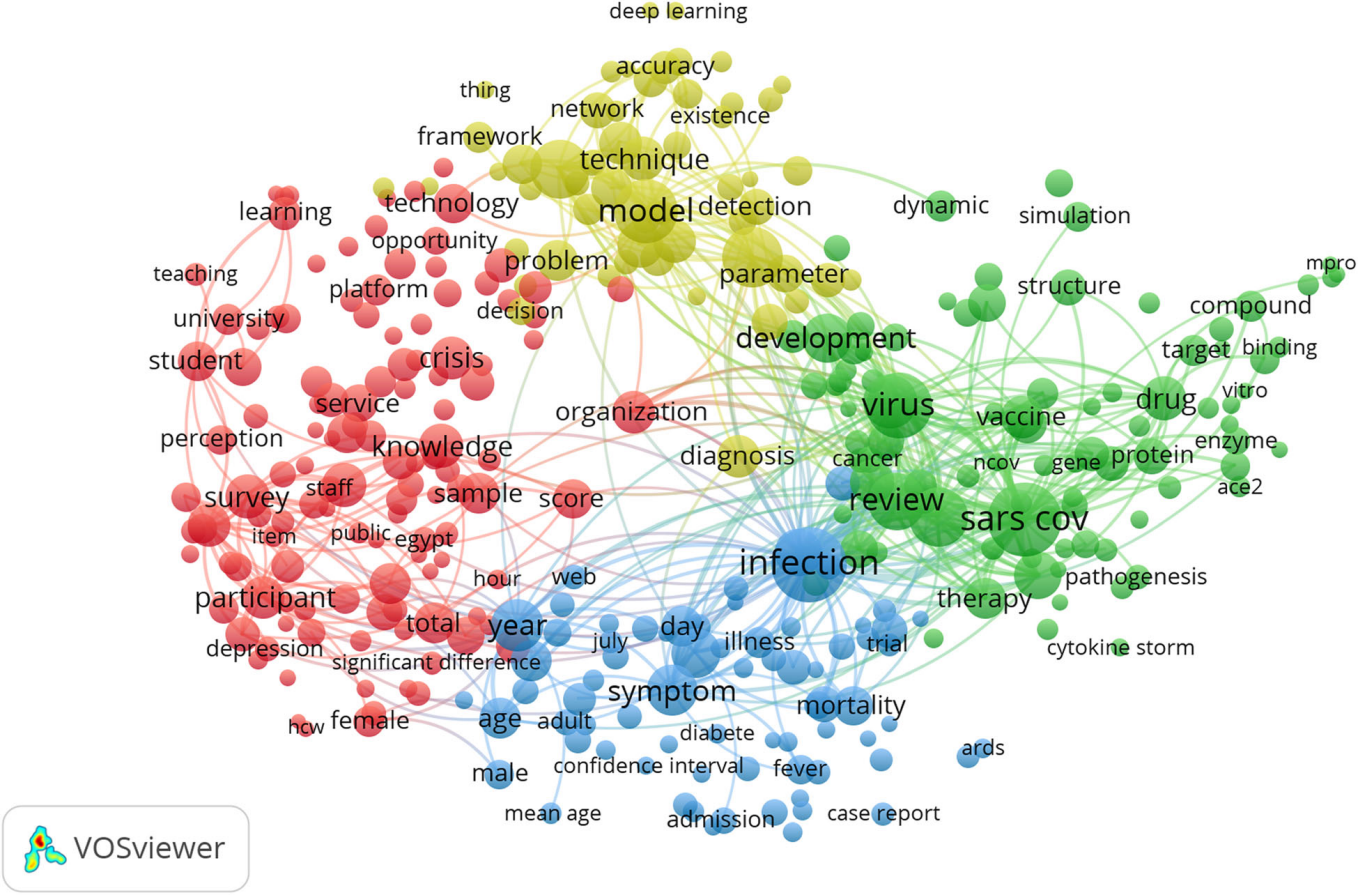
Clustering research topics by mapping Title/Abstract co-occurrences of terms for COVID-19-related publications from the Arab world. Of the 88,868 terms, 570 terms have occurred at least 50 times. A relevance score was calculated for each of the 570 terms and used to choose the 60% most appropriate terms (342 terms). This set of 342 terms was scattered into four different clusters: public health and epidemiology studies (red), immunological and pharmaceutical studies (green), signs, symptoms and clinical diagnosis studies (blue) and virus detection studies (yellow). The map was created using VOSviewer software version 1.6.16

Hot research topics in COVID-19 publications from the Arab world have been presented in network visualization by mapping more than 50 times the co-occurrence of terms in the Title/Abstract in Scopus database publications (Fig. 2). A total of 342 out of the 88,868 terms reached the threshold and this set of terms was scattered into four different clusters (Fig. 2). The highest cluster (Cluster 1: red colour) contains 123 terms that refer mainly to public health and epidemiology, such as “perception”, “education”, “knowledge”, “survey”, “student”, “experience” and “practice”. Cluster 2 (green colour) involves 91 terms related to viruses, including immunological and pharmaceutical research, such as “ACE2”, “replication”, “receptor”, “binding”, “cytokine”, “therapy”, “clinical trial”, “vaccine”, “drug” and “interaction”. Cluster 3 (blue colour) involves 73 terms related to signs, symptoms and clinical diagnoses, such as “fever”, “sign”, “admission”, “case report”, “comorbidity”, “mortality”, “acute respiratory distress syndrome” and “diagnosis”. Finally, Cluster 4 (yellow colour) includes 55 terms related to virus detection, such as “detection”, “prediction”, “model”, “diagnosis”, “sensitivity” and “specificity”.

## Discussion

Bibliometric studies provide interesting methods for measuring the scientific value of a particular field over a specific time. This study mapped Arab research in COVID-19 during the early phase of the epidemic. The findings show that research collaboration is overwhelmingly spread between high-income countries/regions and Arab countries.

However, only the top three countries in the Arab world – Saudi Arabia, Egypt and the UAE – ranked worldwide in terms of the number of COVID-19 research publications: 17th, 33rd and 44th, respectively. In the Arab world, the number of publications remains remarkably lower than in the rest of the world, despite much improvement in COVID-19. The bulk of publications often come from a small number of countries (i.e. Saudi Arabia, Egypt and the UAE) and institutions within these countries.

These findings tend to be compatible with other health research systems in the Arab world that have previously been identified in various health areas, such as breast cancer research [67], complementary medicine research [68], dengue research [17], infectious disease research [69], leishmaniasis research [70], mental health research [71], pharmaceutical wastewater research [72], road traffic injuries [73], tobacco smoking research [74] and toxicology research [75]. In general, in recent decades, the amount of medical research conducted in the Arab world has increased dramatically but is still relatively low compared to other countries in the world [76]. Lack of sufficient scientific infrastructure and services, lack of resources, political instability in Arab countries and difficulty publishing in high-impact journals are reasons for this shortcoming [77–80]. With regard to COVID-19 publications, the open access policy introduced by many publishers has theoretically led to the accelerated distribution of information and the explosive growth of publications over a short time [25].

A large amount of meaningful data can be obtained from the study of term co-occurrence, allowing hotspots and patterns to be identified and directing researchers to relevant topics in their field [81, 82]. Therefore, the main research lines identified in COVID-19 from the Arab world in the current study are related to: public health and epidemiology; immunological and pharmaceutical research; signs, symptoms and clinical diagnosis; and virus detection. Previous studies at the global level [11, 31, 32] have shown the same findings in research directions, in line with the current results. A global bibliometric analysis of COVID-19 conducted by Deng et al. [11] found that four research areas covered the principal topics of public health, clinical and pharmaceutical research and preventive medicine and epidemiology. Zyoud and Al-Jabi [32] performed another global bibliometric review of COVID-19. They shed fresh light on the main progress of hot research topics on COVID-19, including studies of clinical characteristics, pathological findings, therapeutic design, planning of care facilities and infection control. In fact, Arab countries, like many others, have been highly collaborative in science, hitting 50.9% for COVID-19, which is a potential reason for this similarity between Arab countries and the global level. Continuing and improving future collaboration between developing and developed countries will also help to facilitate the sharing of responsibilities for COVID-19 in research results and the implementation of policies for COVID-19.

The article with the highest total citations (*n* = 940) was published by Hui et al. [47] in the *International Journal of Infectious Diseases* as an editorial and concluded that the exchange of knowledge and learning from all geographical regions and across disciplines would be necessary to maintain and further improve development. The second top-cited article was published in *Travel Medicine and Infectious Disease* as a systematic review and meta-analysis [48] and concluded that because this coronavirus has spread globally, human resources, infrastructure and facilities need to be prepared urgently for each country to treat extreme COVID-19 cases. In addition, the third top-cited article was published in *The Lancet* as a systematic review and meta-analysis [49] and recommended that protection is strongly correlated with physical distances of at least 1 m apart but distances of up to 2 m may be more efficient. Furthermore, the fourth top-cited article was published in *Intensive Care Medicine* as a guideline [50] and found that 54 statements were released by the Surviving Sepsis Campaign COVID-19 panel (including 4 best practice statements, 9 strong recommendations and 35 poor recommendations). In addition, the research highlighted in the most widely cited publications introduces the main current hot topics in the current study [47–66], which offers a substantial and valuable perspective on growing publications and hot topics in this field that motivate research development over time. The top-cited articles’ essential contribution is that it would be necessary to exchange knowledge and to learn from all geographical areas and across disciplines to maintain and further improve the progress achieved.

### Strengths and limitations

To the best of our knowledge, this study was the first to conduct a bibliometric analysis of the documentary records of COVID-19-related research during the early phase of the epidemic by using VOSviewer to assess the current hot topics of Arab world research based on COVID-19. The present bibliometric analysis has some limitations and constraints. For instance, because the Scopus database is considered the most accurate and comprehensive database of publications and citations, PubMed and Web of Science were not included in the analyses. No search query is 100% perfect and there is always a chance for false positives and false negatives [83]: databases such as PubMed, EMBASE, Web of Science, Google Scholar and Dimension may give different sets of records for searching but a comparison is beyond the scope of bibliometric analysis in the current study. In our research, the ranking of institutions presented was based on the data given by Scopus. The names of institutions may vary in spelling in some instances, which could generate an inaccuracy in these institutions’ recorded productivity. In addition, owing to the brief amount of time after the pandemic initiation and the continually shifting existence of COVID-19 research, the number of citations will vary with time.

## Conclusions

The following conclusions are drawn based on the findings of this bibliometric research: (1) countries such as Saudi Arabia and Egypt, considering the skills of Saudi and Egyptian scientists in this area, can lead Arab researchers in this field; (2) with the global upsurge in COVID-19 study, substantial collaborations have been established between countries or regions, among which the USA and the UK are at the centre of cooperation and have the strongest relations of cooperation with Arab countries; and (3) In the current analysis, the key research lines found in COVID-19 from the Arab world are related to: public health and epidemiology; immunological and pharmaceutical research; signs, symptoms and clinical diagnosis; and virus detection. The outcomes of the current study will enable researchers, academics, clinicians and government leaders from the Arab world to enhance efficiency in future studies and understand further applications in the area of COVID-19. In particular, understanding the evolution of the emerging scientific knowledge on COVID-19 is beneficial not just to the scientific community but also to evidence-based policy-making to fully resolve the COVID-19 pandemic’s implications. To introduce and carry out research on COVID-19, researchers in low- and middle-income countries such as the Arab world must develop collaborations and connections with researchers in high-income countries.

## Data Availability

All data generated or analysed during this study are included in this published article. Other datasets used during the current study are available from the author on reasonable request (saedzyoud@yahoo.com).

## Abbreviations

AI: Adjustment index
COVID-19: Coronavirus disease 2019
GDP: Gross domestic product
UAE: United Arab Emirates
WHO: Orld Health Organization
